# Cumulative Vascular Burden: The Prognostic Value of a Time-Weighted Cardio-Ankle Vascular Index in Haemodialysis Patients

**DOI:** 10.1159/kbr/adjag007

**Published:** 2026-09-07

**Authors:** Roberto Casale, Lida Tartaglione, Francesco Pesce, Sandro Mazzaferro, Silvia Lai, Sebastiano Sciarretta, Maria Luisa Muci, Antonio Bellasi, Gianluigi Zaza, Silverio Rotondi

**Affiliations:** a Division of Renal Medicine, Ospedale Isola Tiberina, Gemelli Isola, Rome, Italy; b Université libre de Bruxelles (ULB), Hôpital Universitaire de Bruxelles (HUB), Institut Jules Bordet, Brussels, Belgium; c Renal Division, Umberto I Policlinico di Roma, Rome, Italy; d Department of Translational Medicine and Surgery, Università Cattolica del Sacro Cuore, Rome, Italy; e Department of Translational and Precision Medicine, Università degli Studi di Roma La Sapienza, Rome, Italy; f Medical and Surgical Biotechnological Sciences, Università degli Studi di Roma La Sapienza, Rome, Italy; g Division of Nephrology, Ente Ospedaliero Cantonale, Ticino, Switzerland

**Keywords:** Arterial stiffness, Cardio-ankle vascular index, Cumulative vascular burden, Haemodialysis, Survival analysis

## Abstract

**Introduction:**

Chronic kidney disease and end-stage renal disease are burdened by extremely high cardiovascular mortality. The cardio-ankle vascular index (CAVI) is a blood-pressure-independent marker of arterial stiffness, but a single measurement may not reflect the time-dependent nature of vascular remodelling in haemodialysis. We hypothesized that a time-weighted CAVI (twCAVI), integrating arterial stiffness with dialysis vintage, would better predict all-cause mortality.

**Methods:**

In this observational cohort study, 94 maintenance haemodialysis patients were followed from February 2016 to December 2024. Arterial stiffness was assessed by CAVI, and twCAVI was defined as mean CAVI × dialysis vintage (months). After preprocessing and feature selection, the association between arterial stiffness and overall survival over approximately 8 years was examined using a Cox proportional hazards model. Internal validity was assessed by 1,000 bootstrap iterations and expressed via Harrell’s concordance index (C-index) and time-dependent area under the curve (AUC).

**Results:**

The final model retained six predictors: sex, vascular access, creatinine, alkaline phosphatase (ALP), ferritin, and twCAVI. In the multivariate model, male sex (HR 2.56, *p* = 0.0018), ALP (HR 1.01, *p* = 0.0082), ferritin (HR 1.002, *p* = 0.0067) and twCAVI (HR 1.0021, *p* = 0.0464) were independently associated with higher mortality, while higher creatinine was protective (HR 0.87, *p* = 0.0445). Central venous catheter use showed a borderline trend towards increased risk compared with arteriovenous fistula (HR 1.68, *p* = 0.0509). Bootstrap validation yielded a median C-index of 0.75 (95% confidence interval [CI] 0.68–0.83), with time-dependent AUCs generally ≥0.80 up to 102 months. Notably, exploratory survival analyses revealed an age-dependent inverse association between phosphorus levels and mortality, supporting the reverse epidemiology hypothesis in the elderly.

**Conclusion:**

twCAVI, capturing the cumulative vascular burden risk, is a predictor of mortality in haemodialysis patients and improves long-term risk discrimination. A proof-of-concept web-based calculator was developed to facilitate the educational exploration of this metric.

## Introduction

Chronic kidney disease (CKD) and end-stage renal disease (ESRD) represent a growing global health burden, significantly associated with elevated cardiovascular (CV) mortality. Despite advancements in dialysis technology and pharmacological management, CV disease remains the leading cause of death in this population. A hallmark of CV pathology in dialysis patients is arterial stiffness, a consequence of medial vascular calcification (Mönckeberg’s sclerosis) [1], endothelial dysfunction, and chronic volume overload [2]. Consequently, the assessment of arterial stiffness has become a key factor in stratifying CV risk in the dialysis population [3, 4].

Historically, carotid-femoral pulse wave velocity (PWV) has been considered the gold standard for measuring arterial stiffness [5]. However, PWV is inherently dependent on blood pressure at the time of measurement, which can introduce bias in haemodialysis patients. In this population, pre- and post-dialysis blood pressure values show marked variability, largely reflecting fluctuations in volume status related to interdialytic fluid accumulation and intradialytic fluid removal [6, 7], thereby potentially confounding standard arterial stiffness assessments. In this context, the cardio-ankle vascular index (CAVI) has been proposed in patients undergoing haemodialysis as a more reliable indicator of arterial stiffness [8, 9]. Derived from the stiffness parameter *β*, CAVI represents a pressure-independent measure of arterial stiffness, theoretically reflecting the structural properties of the arterial wall from the origin of the aorta to the ankle [10, 11]. Several studies have demonstrated that high CAVI scores are predictors of CV events in CKD patients [12].

However, the interpretation of arterial stiffness in the specific context of haemodialysis requires a nuanced approach that accounts for the duration of time a patient has been exposed to renal replacement therapy. The progression of vascular remodelling in ESRD is highly heterogeneous. In the early stages of dialysis, arterial elasticity may still be preserved, and a rapid increase in stiffness (CAVI) may represent a distinct, aggressive pathological phenotype with high prognostic significance. Conversely, a long dialysis vintage chronically exposes patients to systemic inflammation, altered mineral bone metabolism, and associated malnutrition. Over time, these interconnected factors drive diffuse vascular calcification. This extensive calcification leads to a “ceiling effect” where stiffness is uniformly elevated, potentially reducing the discriminative power of a single static CAVI measurement [13]. A similar paradox is well-documented with other haemodynamic markers, such as the Ankle-Brachial Index; in end-stage renal disease, severe arterial calcification makes the arteries incompressible, leading to abnormally high measurements that reflect a high CV risk but simultaneously lose the ability to detect underlying occlusive stenotic disease. Therefore, a static measurement of CAVI might suffer from a similar saturation, failing to capture the cumulative and dynamic impact of arterial stiffening over time.

We postulate that the prognostic power of CAVI is modulated by the duration of dialysis exposure. To test this hypothesis, we developed a novel interaction metric, the time-weighted CAVI (twCAVI), calculated as the product of the mean CAVI and dialysis vintage (the time from dialysis initiation to measurement). This composite index is designed to reflect the “cumulative vascular burden” by integrating the severity of arterial stiffness over the duration of dialysis exposure.

The primary objective of this study is to evaluate the association between this novel time-weighted metric and all-cause mortality in a cohort of haemodialysis patients. Furthermore, we aimed to develop a multivariate all-cause survival prediction model using feature selection techniques and bootstrap validation. To facilitate clinical translation, the resulting model has been implemented in a web-based application, enabling personalized risk stratification.

## Materials and Methods

### Study Design and Population

This was a retrospective, observational, longitudinal cohort study involving 94 patients undergoing maintenance haemodialysis. The observation period extended from February 1, 2016, to December 31, 2024, covering a total of 107 months. Patients were recruited from the Nephrology unit at Polo Pontino, Sapienza University of Rome. Inclusion criteria comprised patients aged >18 years with a diagnosis of ESRD on chronic haemodialysis for at least 1 month. Patients were censored at the time of kidney transplantation, or loss to follow-up. The follow-up time was calculated as the interval between the date of CAVI assessment and the date of death or censoring.

### Clinical and Laboratory Assessment

Baseline demographic and clinical data were collected at the time of vascular assessment. Recorded variables included age, sex, and vascular access type (central venous catheter [CVC] vs. arteriovenous fistula [AVF]). A comprehensive panel of biochemical parameters was assessed, including haemoglobin, haematocrit, urea, creatinine, total protein, albumin, inflammatory markers (C-reactive protein [CRP], erythrocyte sedimentation rate [ESR], fibrinogen, ferritin, transferrin). Markers of bone-mineral metabolism were evaluated, including calcium, magnesium, alkaline phosphatase (ALP), phosphorus (P), and parathyroid hormone (PTH). Given the non-linear, U-shaped association between mortality and both phosphorus and PTH levels in dialysis populations, these variables were dichotomized based on traditional clinical targets and established mortality risk associations [14]. Specifically, Phosphorus was categorized as “Low Risk” (3.5–5.5 mg/dL) versus “High Risk” (<3.5 or >5.5 mg/dL). Similarly, PTH was dichotomized as “Low Risk” (150–300 pg/mL) versus “High Risk” (<150 or >300 pg/mL) to capture both low-turnover bone disease and secondary hyperparathyroidism.

Haemodynamic parameters, including systolic and diastolic blood pressure, were measured both pre- and post-dialysis. Arterial stiffness was assessed using the CAVI (Fukuda VaSera VS-1500). The mean CAVI was calculated as the average of the right and left limb measurements. Similarly, mean pulse wave velocity (PWV) was recorded. The augmentation index (AI) was measured to assess peripheral arterial wave reflection. To account for the interaction between arterial stiffness and the duration of disease exposure, we introduced a composite variable defined as:

$$\text{twCAVI}={\text{CAVI}}_{\text{mean}}\times \text{DialysisTiming}\;\left(\text{vintage}\right)$$

where “Dialysis Timing” is the time (in months) from dialysis inception to the moment of CAVI measurement. Considering that the cardio-ankle vascular index (CAVI) is a unitless measure used to assess arterial stiffness, likewise, twCAVI represents a cumulative, time-weighted index.

### Statistical Analysis

#### Data Preprocessing

Missing values for continuous variables were imputed using the median strategy, while categorical variables were imputed using the most frequent category. Continuous variables were not standardized to preserve clinical interpretability. Categorical variables (sex, vascular access) were transformed into a numerical format suitable for modelling via One-Hot Encoding. This standard technique generates binary dummy variables, preventing the statistical model from misinterpreting these non-ordinal features as continuous or ranked data. Crucially, one category within each variable was omitted from the encoding process to prevent multicollinearity (redundancy) in the model.

#### Feature Selection Pipeline

To construct a robust and parsimonious model, we first excluded the base variables CAVI and Dialysis Timing from the analysis. This a priori step prevents structural multicollinearity, as these variables constitute the direct mathematical components of the twCAVI metric (twCAVI = CAVI × Dialysis Timing). Subsequently, we applied a three-step feature selection process:

1. Multicollinearity Reduction: We calculated the Spearman correlation matrix. For pairs of features with a correlation coefficient >0.80, the variable with the lower variance was removed to reduce redundancy while retaining information-rich features [15, 16].
2. Univariate Cox Screening: We performed univariate Cox regression for all significant variables identified [17]. Features with *p* values <0.25 were retained in the multivariate stage. This threshold is recommended to prevent excluding variables that may become significant only in a multivariate context [18].
3. Stepwise Backward Elimination: starting from the full model that included all features retained in the univariate analysis, a stepwise backward selection was carried out to identify the most parsimonious model to predict survival. Variables were removed iteratively if their associated *p* value exceeded the predetermined cut-off of 0.25 [18, 19].

#### Survival Analysis

The final predictive model was built using Cox Proportional Hazards Regression. We employed robust standard errors for parameter estimation, utilizing the lifelines library implementation (robust = True) [20]. As noted by Stensrud and Hernán, strict adherence to the proportional hazard’s assumption is often unrealistic in biological datasets [21]. The use of robust standard errors allows the estimated hazard ratios to be interpreted as time-weighted averages of the covariate effects, maximizing predictive utility even if the assumption is strictly violated [20].

Finally, to elucidate the prognostic role of phosphorus and investigate potential effect modification by age, an exploratory stratified analysis was conducted. The cohort was divided into two subgroups based on the median age (73 years). Separate Cox proportional hazards models were fitted for the categorized phosphorus variable (“Low Risk” vs. “High Risk”) within each stratum (≤73 years and >73 years) to verify if the association with mortality differed between younger and older patients.

#### Model Validation

We performed a non-parametric bootstrap validation with 1,000 iterations to assess the internal validity and stability of the model [22, 23]. In each iteration, the dataset was resampled with replacement, and the apparent performance of the model was evaluated on the resampled data. Performance metrics included the concordance index (C-index) [24], and time-dependent area under the receiver operating characteristic curve (AUC) at 6-month intervals from month 6 to 102. Optimal cut-off points for risk classification were determined using the Youden index (sensitivity + specificity − 1) [25].

All analyses were performed using Python (v3.12), utilizing the lifelines library for survival analysis [20] and scikit-learn for preprocessing and metrics [26]. A significance level of *p* < 0.05 was considered statistically significant for the final model coefficients.

The final model was deployed as a client-side web application using React.js. To ensure data privacy and prevent re-identification risks consistent with k-anonymity principles [27], model parameters were rounded as a generalization measure. This architecture ensures that no patient data is transmitted to external servers. The source code is publicly available for transparency and reproducibility at https://github.com/roberto-casale/CAVI_Web_App.

### Use of Artificial Intelligence

Large language models were used as assistive tools during the preparation of this work. Specifically, Google Gemini (Google LLC) was used to support language refinement and to provide technical assistance during the development of the web-based calculator and of the statistical analysis pipeline; Claude (Anthropic) was used for language refinement, manuscript formatting and consistency checks during preparation for submission. All outputs produced with the assistance of these tools were directly supervised, verified and, where appropriate, revised by the authors, who take full responsibility for the accuracy, integrity, and originality of the final work.

## Results

### Baseline Characteristics and Feature Selection

The study population consisted of 94 patients. Following the removal of identifiers, the dataset comprised demographic, biochemical, and haemodynamic features. Table 1 summarizes the baseline characteristics of the study cohort, detailing continuous and categorical variables along with their respective units of measurement, including the outcome status.

**Table 1.** Baseline characteristics and outcome status of the study cohort

Feature	Mean±SD	Median [IQR]
Age	69.95±14.50	73.00 [62.50–80.75]
PWV, mean	8.74±2.01	8.34 [7.75–9.07]
AI	1.03±0.21	1.00 [0.91–1.06]
SAP_pre, mm Hg	135.38±14.76	134.98 [125.55–144.78]
DAP_pre, mm Hg	71.24±10.11	71.00 [64.31–77.20]
SAP_post, mm Hg	135.85±14.75	135.74 [126.00–147.30]
DAP_post, mm Hg	73.00±9.84	72.94 [68.00–79.32]
BMI, kg/m^2^	25.52±5.53	24.45 [21.50–28.85]
Urea, mg/dL	64.67±12.89	65.15 [58.18–72.87]
Creatinine, mg/dL	9.04±1.97	8.97 [7.81–9.99]
Mg, mg/dL	2.08±0.33	2.08 [1.85–2.30]
Ca, mg/dL	8.58±0.52	8.56 [8.35–8.94]
Total protein, g/dL	6.35±0.46	6.34 [6.20–6.50]
Alb, g/dL	3.43±0.31	3.42 [3.30–3.56]
ALP, U/L	77.30±23.80	72.88 [62.19–84.75]
Hb, g/dL	10.48±0.98	10.45 [9.96–11.16]
Hct, %	32.63±2.80	32.58 [30.93–34.59]
CRP, mg/dL	1.45±1.81	0.80 [0.50–1.45]
ESR, mm/h	32.22±18.62	27.65 [19.93–39.00]
Fib, mg/dL	321.46±61.27	319.73 [284.00–344.51]
Ferritin, ng/mL	275.41±183.49	272.15 [132.60–352.50]
Transferrin, mg/dL	188.11±42.74	184.50 [166.50–203.91]
Follow-up time	54.73±36.34	43.50 [23.25–100.00]
twCAVI	175.71±160.01	125.78 [66.15–250.60]
Sex, *n* (%)	f: 44 (46.8)	​
​	m: 50 (53.2)	​
Vascular access, *n* (%)	AVF: 67 (71.3)	​
​	CVC: 27 (28.7)	​
Phosphorus status, *n* (%)	Low Risk: 61 (64.9)	​
​	High Risk: 33 (35.1)	​
PTH status, *n* (%)	Low Risk: 34 (36.2)	​
​	High Risk: 60 (63.8)	​
Event, *n* (%)	0 (Censored): 26 (27.7)	​
​	1 (Dead/Event): 68 (72.3)	​

Continuous variables are presented as Mean ± SD and Median [Interquartile Range (IQR)], while categorical variables are reported as frequency (percentage).

The feature selection process significantly reduced dimensionality. Initially, highly correlated features such as haemoglobin (correlated with haematocrit) and post-dialysis diastolic pressure (DAP_post) were removed (Spearman *p* > 0.8). The subsequent univariate Cox screening (*p* < 0.25) and stepwise backward elimination identified a final set of 6 independent predictors: sex, vascular access, creatinine, ALP, ferritin, and twCAVI.

### Univariate and Multivariate Cox Regression Analysis

The univariate cox proportional hazards analysis is summarized in Table 2; in accordance with the described analysis, phosphorus and PTH were included in the univariate models as a categorical variable rather than a continuous feature. The subsequent multivariate cox proportional hazards model demonstrated a robust fit; the results of the final model are summarized in Table 3.

**Table 2.** Univariate Cox proportional hazards analysis

Feature	Coef	HR	HR lower 95%	HR upper 95%	*p* value
Age	0.0223	1.0226	1.0054	1.0401	0.0100
ALP	0.0106	1.0107	1.0025	1.0189	0.0102
Alb	−0.5282	0.5896	0.2740	1.2690	0.1768
AI	0.2183	1.2440	0.5091	3.0395	0.6319
BMI	−0.0226	0.9776	0.9381	1.0188	0.2823
Ca	−0.0314	0.9691	0.6402	1.4671	0.8822
Creatinine	−0.1052	0.9001	0.7965	1.0171	0.0915
DAP_post	−0.0200	0.9802	0.9601	1.0008	0.0598
DAP_pre	−0.0272	0.9732	0.9521	0.9948	0.0152
Ferritin	0.0017	1.0017	1.0001	1.0033	0.0360
Fib	0.0024	1.0024	0.9984	1.0064	0.2399
Hb	−0.1087	0.8970	0.6896	1.1667	0.4177
Hct	−0.0066	0.9935	0.9108	1.0836	0.8822
Mg	−0.1021	0.9029	0.4839	1.6849	0.7484
Phosphorus status	−0.5080	0.6017	0.3506	1.0326	0.0652
CRP	0.0931	1.0975	1.0081	1.1949	0.0318
PTH status	0.2659	1.3046	0.7957	2.1389	0.2919
PWV, mean	0.0204	1.0206	0.8923	1.1673	0.7663
SAP_post	0.0038	1.0038	0.9884	1.0194	0.6315
SAP_pre	0.0038	1.0038	0.9864	1.0215	0.6701
Male sex	0.4928	1.6368	1.0068	2.6611	0.0469
Transferrin	−0.0005	0.9995	0.9944	1.0047	0.8514
Total protein	−0.0073	0.9928	0.6136	1.6063	0.9764
twCAVI	0.0017	1.0017	0.9997	1.0037	0.0889
Urea	−0.0082	0.9918	0.9724	1.0116	0.4161
Vascular_access	0.7165	2.0472	1.2498	3.3535	0.0044
ESR	0.0102	1.0103	0.9994	1.0212	0.0641

Hct, haematocrit.

Hazard ratios (HRs), calculated as exp(coef), and 95% confidence intervals.

**Table 3.** Multivariate Cox proportional hazards analysis

Feature	Coef	HR	HR lower 95%	HR upper 95%	*p* value
ALP	0.0109	1.0109	1.0028	1.0191	0.0082
Creatinine	−0.1389	0.8703	0.7600	0.9966	0.0445
Ferritin	0.0019	1.0019	1.0005	1.0033	0.0067
Male sex	0.9405	2.5612	1.4186	4.6243	0.0018
twCAVI	0.0021	1.0021	1.0000	1.0043	0.0464
Vascular access (CVC)	0.5163	1.6758	0.9980	2.8138	0.0509

Hazard ratios (HRs), calculated as exp(coef), and 95% confidence intervals.

Male sex emerged as the strongest predictor of mortality (HR 2.56, *p* = 0.0018). Elevated levels of ALP (HR 1.01, *p* = 0.0082) and ferritin (HR 1.002, *p* = 0.0067) were also confirmed as significant independent risk factors. Conversely, higher serum creatinine was identified as a protective factor (HR 0.87, *p* = 0.0445). Regarding vascular access, the use of a CVC showed a notable trend towards increased risk compared to AVF, although it just failed to reach statistical significance (HR 1.68, *p* = 0.0509). Most importantly, the novel metric twCAVI proved to be a significant independent predictor of mortality (HR 1.0021, *p* = 0.0464), suggesting that cumulative vascular burden provides additional prognostic value beyond standard haemodynamic parameters. Although the unit-based hazard ratio appears small (1.0021), the clinical impact is substantial given the wide range of twCAVI values (IQR 66.15–250.60). For instance, an increment of 100 units, biologically equivalent to one additional year on dialysis (12 months) with a mean CAVI of 8.3, is associated with a 23% increase in mortality risk (1.0021^(12*8.3)^ ≈ 1.23).

### Age-Stratified Analysis of Phosphorus Status

In the overall univariate analysis, Phosphorus status showed a counterintuitive trend indicating a protective effect for the “High Risk” category (HR 0.60, *p* = 0.065). To better understand how phosphorus and age interact to affect overall survival, we conducted a stratified analysis. Stratifying the study cohort by median age (73 years) revealed distinct differences in risk profiles between younger and older patients. In the elderly subgroup (>73 years, *n* = 45), the “High Risk” status was associated with a marked reduction in mortality risk (HR 0.41, 95% CI: 0.15–1.17), although this did not achieve statistical significance (*p* = 0.097). Conversely, in the younger subgroup (≤73 years, *n* = 49), Phosphorus status was not associated with mortality (HR 0.93, *p* = 0.84). This observation suggests that the “protective” signal detected in the general cohort is primarily driven by the elderly population.

### Independence of Selected Features

Spearman correlation analysis of the final selected features confirmed the absence of significant multicollinearity (Fig. 1), ensuring the stability of the coefficients. The maximum absolute correlation observed between predictors was 0.32, confirming that the selected predictors provide distinct information.

**Fig. 1.**
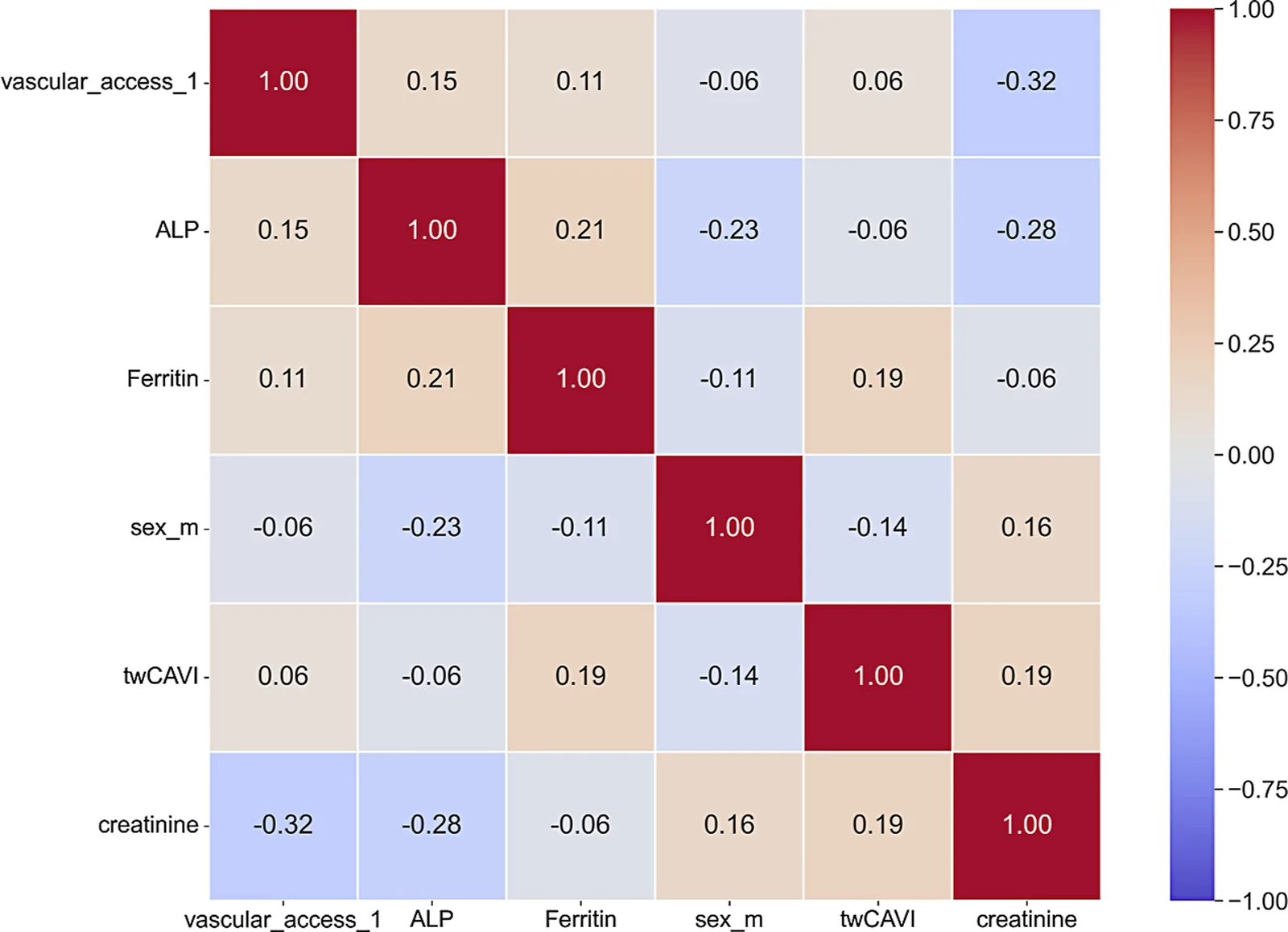
Spearman correlation analysis of the final selected features.

### Model Validation and Performance

The internal validity of the model was tested using 1000 bootstrap iterations. The global median C-index was 0.75 (95% CI: 0.68–0.83), indicating good discriminatory power.

Time-dependent performance analysis revealed consistent predictive accuracy across the follow-up period (Table 4). The median AUC remained above 0.80 for most time points.

**Table 4.** Time-dependent predictive performance

Time (months)	AUC (95% CI)	Sensitivity (95% CI)	Specificity (95% CI)	Accuracy (95% CI)
6	0.80 (0.48–0.95)	0.90 (0.50–1.00)	0.76 (0.30–1.00)	0.77 (0.34–0.97)
12	0.81 (0.57–0.94)	0.86 (0.45–1.00)	0.77 (0.35–0.99)	0.79 (0.43–0.95)
18	0.85 (0.66–0.95)	0.83 (0.50–1.00)	0.85 (0.49–1.00)	0.84 (0.56–0.96)
24	0.80 (0.65–0.92)	0.75 (0.44–1.00)	0.83 (0.42–1.00)	0.80 (0.56–0.90)
30	0.82 (0.68–0.93)	0.74 (0.48–0.97)	0.86 (0.49–1.00)	0.81 (0.63–0.90)
36	0.82 (0.70–0.91)	0.75 (0.49–0.97)	0.83 (0.49–1.00)	0.79 (0.66–0.87)
42	0.81 (0.69–0.89)	0.74 (0.45–0.98)	0.81 (0.47–1.00)	0.77 (0.67–0.86)
48	0.81 (0.69–0.90)	0.75 (0.47–0.98)	0.80 (0.47–1.00)	0.77 (0.66–0.86)
54	0.82 (0.71–0.90)	0.76 (0.49–0.97)	0.82 (0.50–1.00)	0.77 (0.67–0.86)
60	0.83 (0.73–0.92)	0.78 (0.52–0.97)	0.82 (0.52–1.00)	0.79 (0.68–0.87)
66	0.84 (0.73–0.92)	0.80 (0.53–0.98)	0.81 (0.54–1.00)	0.80 (0.67–0.88)
72	0.83 (0.73–0.92)	0.80 (0.54–0.98)	0.81 (0.53–1.00)	0.80 (0.67–0.88)
78	0.84 (0.74–0.93)	0.81 (0.56–0.98)	0.82 (0.55–1.00)	0.81 (0.67–0.89)
84	0.85 (0.75–0.93)	0.81 (0.55–0.98)	0.82 (0.56–1.00)	0.81 (0.67–0.90)
90	0.85 (0.75–0.93)	0.81 (0.55–0.98)	0.82 (0.56–1.00)	0.81 (0.67–0.90)
96	0.85 (0.75–0.93)	0.83 (0.56–0.98)	0.82 (0.55–1.00)	0.82 (0.67–0.91)
102	0.89 (0.76–0.97)	0.85 (0.57–0.99)	0.88 (0.62–1.00)	0.86 (0.64–0.96)

Metrics are reported as median values with 95% confidence intervals (in parentheses) derived from 1,000 bootstrap iterations.

The time-dependent predictive performance of the multivariate Cox model was evaluated every 6 months over a follow-up period extending to 102 months. Figure 2 illustrates the trajectory of key performance metrics (AUC, sensitivity, specificity, and accuracy) derived from 1,000 bootstrap iterations. The model demonstrated robust stability across the entire time horizon. The median AUC remained consistently high, ranging between 0.80 and 0.89, indicating strong discriminative ability for both short- and long-term mortality risks. Accuracy showed a similar stable trend, maintaining values near 0.80. Sensitivity and specificity exhibited balanced performance, suggesting the model reliably distinguishes between high-risk and low-risk patients regardless of the prediction window.

**Fig. 2.**
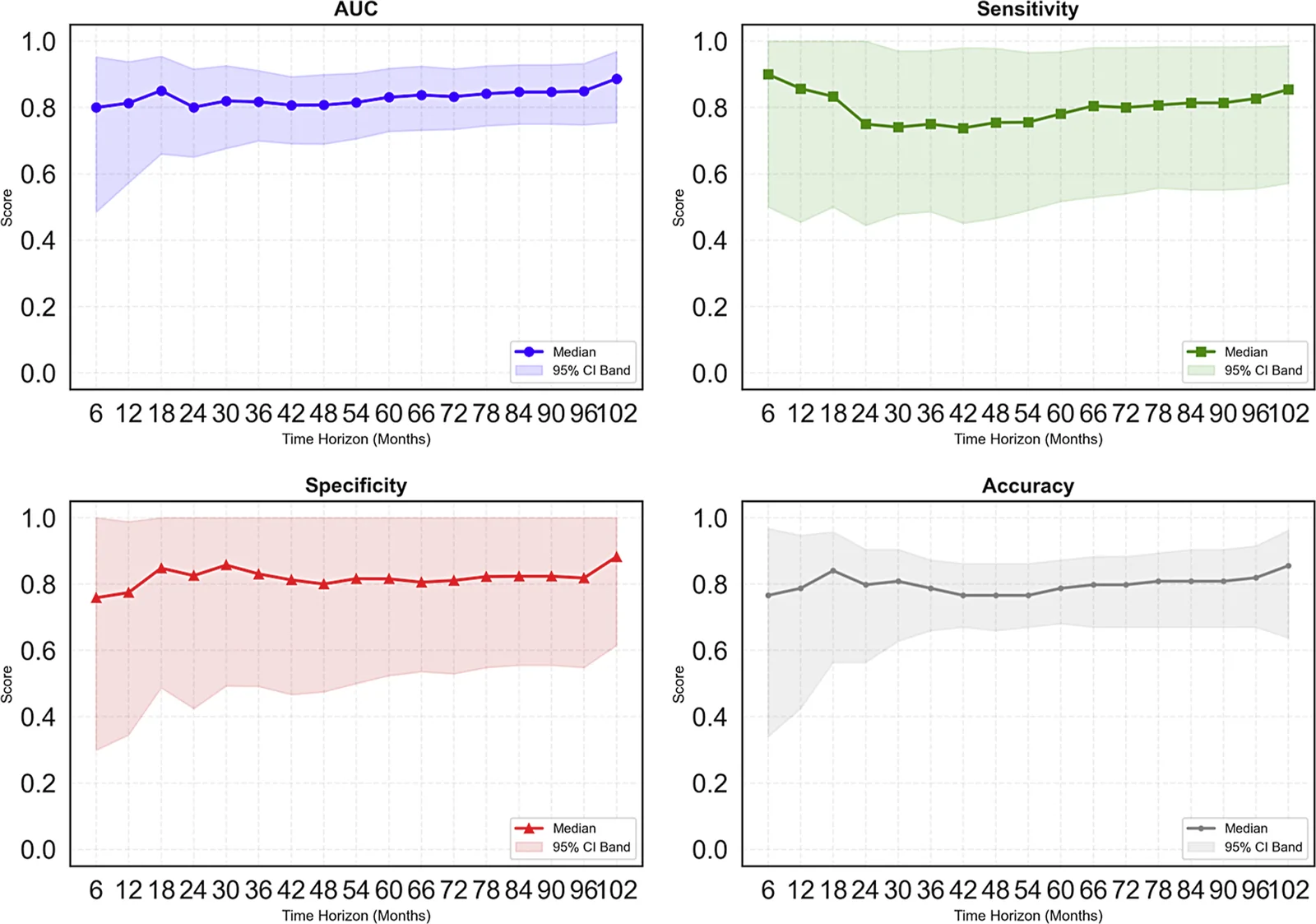
Time-dependent model performance metrics over 102 months. The four panels display the longitudinal trends for Area Under the Curve (AUC), Sensitivity, Specificity, and Accuracy. Solid lines with markers represent the median values calculated.

To provide a tangible proof-of-concept of the multivariate model, a web-based calculator was developed. This tool is intended for educational and demonstrative purposes only and has not yet been validated for clinical decision-making. It allows users to input variables to explore the estimated mortality risk probability (available at: https://cavi-web-app.vercel.app/). The calculator operates entirely offline within the user’s browser to guarantee strict data confidentiality.

## Discussion

The primary objective of this study was to evaluate the prognostic value of a novel composite metric, the time-weighted cardio-ankle vascular index (twCAVI), in a cohort of maintenance haemodialysis patients. Our findings demonstrate that the integration of arterial stiffness assessment with the duration of dialysis exposure significantly improves mortality risk stratification.

The multivariate Cox proportional hazards model identified twCAVI, along with male sex, serum creatinine, ferritin, ALP, and vascular access as independent predictors of all-cause mortality.

The significance of the twCAVI term (HR 1.0021, *p* = 0.0464) highlights an aspect of vascular pathophysiology in ESRD: the time-dependency of arterial damage. While conventional parameters like PWV or static CAVI are well-established predictors of CV events [10], they often fail to capture the historical exposure to the uraemic milieu. In long-term dialysis patients, the vasculature undergoes progressive medial calcification (Mönckeberg’s sclerosis), which can lead to a “stiffness plateau” or ceiling effect, potentially masking acute haemodynamic deterioration [13]. By weighting the CAVI score by dialysis timing, our model accounts for the chronicity of vascular stress. Crucially, while dialysis vintage acts as a dominant mathematical component due to its high variance, the interaction term allows for a vital distinction: it specifically penalizes patients with both prolonged exposure and high stiffness, separating them from the “long survivors” who, despite long-term dialysis, maintain preserved arterial elasticity.

The feature selection process retained variables that reflect the complex “Reverse Epidemiology” often observed in dialysis populations [28, 29]. Higher serum creatinine levels were associated with reduced mortality (HR 0.87, *p* = 0.0445). In the general population, elevated creatinine indicates renal dysfunction; however, in anuric dialysis patients, it serves as a surrogate marker for muscle mass and nutritional status. Consistently, BMI showed a similar protective trend (HR 0.98, *p* = 0.28), further supporting the hypothesis that a better nutritional profile and metabolic reserve confer a survival advantage in this cohort.

Similarly, as observed in our stratified analysis, “High Risk” phosphorus levels demonstrated a protective trend in patients older than 73 years. This supports the “Phosphate Paradigm” proposed by Fouque et al. [29]. In fact, aggressive dietary restrictions to lower phosphate levels can be harmful and lead to protein-energy wasting. Therefore, in this group, higher phosphate levels mainly indicate sufficient protein intake and improved nutritional health, which outweigh the potential toxicity of elevated serum Phosphorus. Importantly, demonstrating this reverse epidemiology quantitatively using a specific age cut-off, particularly within a model that also incorporates cumulative arterial stiffness (twCAVI), represents an original observation. While this age-dependent interaction was not incorporated into the primary predictive model to avoid data-driven bias, this specific finding deserves greater emphasis and strongly warrants dedicated prospective investigations in larger cohorts to refine future iterations of risk assessment tools.

The inclusion of ALP, a marker of high-turnover bone disease and vascular calcification, further refines the model’s ability to detect patients with advanced vascular ossification [30]. Elevated serum ALP, especially its bone-specific isoform, has consistently been linked to ongoing vascular calcification in patients with chronic kidney disease [31, 32]. Although traditionally seen as a marker of bone turnover, this enzyme also seems to play an active role in vascular wall mineralization by hydrolysing pyrophosphate, a strong inhibitor of calcification. Including ALP in our survival model reinforces the idea that bone-mineral imbalances and vascular ageing are mechanistically connected in the dialysis population. This highlights the systemic nature of CKD-mineral and bone disorder, extending beyond bone health to include CV issues.

Ferritin emerged as a significant risk factor (*p* = 0.0067); in the dialysis context, hyperferritinemia is frequently a marker of systemic inflammation [33]. In fact, although ferritin is commonly measured to guide iron supplementation in haemodialysis patients, it also acts as an acute-phase reactant that increases during inflammatory states. Its elevation has been linked to higher mortality, indicating that, beyond reflecting iron stores, ferritin may also capture the harmful prognostic effects of chronic inflammation and improve the model discrimination of risk of all-cause mortality in this group [33].

Male sex was the strongest predictor of mortality (HR: 2.56), a finding universally reproducible in dialysis cohorts [34]. Furthermore, the use of a CVC showed a trend toward increased risk compared to AVF (HR: 1.68). Although strictly non-significant in the final step (*p* = 0.0509), its retention in the model is clinically justified, as CVC use is associated with systemic inflammation, infection, and lower dialysis adequacy compared to fistulas [35].

Our study has several limitations. First, the sample size (*N* = 94); however, we mitigated the risk of overfitting by employing robust feature selection techniques (Stepwise Cox) and Bootstrap validation (1,000 iterations), which confirmed the stability of the C-index (0.75). Second, the observational and retrospective design precludes establishing causality; although twCAVI is associated with mortality, it remains unclear whether therapeutic interventions targeting arterial stiffness can reduce this risk [36]. Third, the study did not account for time-varying covariates; biochemical parameters and CAVI were measured at baseline; however, dialysis patients experience fluctuations in mineral metabolism and haemodynamic status over time. Fourth, regarding sex-stratified analyses, we acknowledge that our overall sample size is underpowered to conduct robust independent survival models for males and females. However, sex was rigorously accounted for in the global multivariate model, where male sex emerged as a strong independent predictor of mortality (HR 2.56, *p* = 0.0018). Fifth, CAVI assessment requires a dedicated device that is not routinely available in most dialysis units, which may currently limit the widespread implementation of twCAVI in real-world clinical practice. However, studies such as the present one are useful to clarify the function and the real clinical utility of this index, providing the rationale for its potential future integration into routine dialysis care.

From a clinical perspective, the identification of an elevated twCAVI indicates a heightened risk of mortality, independent of other traditional risk factors. Therefore, integrating this index into routine clinical practice could be valuable. Specifically, patients with a high twCAVI might benefit from a stricter, highly personalized monitoring of their overall clinical status compared to those with lower scores. In these individuals, an elevated twCAVI could serve as a trigger to proactively reassess other modifiable risk factors. For instance, it might prompt clinicians to adopt personalized strategies to better control volume status, manage systemic inflammation (such as addressing high ferritin levels), and optimize mineral and bone metabolism. Furthermore, in the presence of a high cumulative vascular burden, clinicians might consider prioritizing a shift in vascular access type, potentially favouring the transition from CVC to AVF whenever feasible. Given the observational nature of our study, it remains to be determined whether these tailored interventions can directly improve survival; however, a high twCAVI score may reasonably justify a more intensive and vigilant clinical approach.

To fully realize this clinical potential, future research should focus on validating the twCAVI metric in multi-centre, large-scale cohorts to establish standardized cut-off values. Additionally, the integration of this metric into the developed web-based application provides a platform for educational and demonstrative purposes.

In conclusion, this study introduces the time-weighted cardio-ankle vascular index (twCAVI) as a novel predictor of mortality in haemodialysis patients. The generated predictive model, grounded in the pathophysiological framework of reverse epidemiology and mineral bone disorder, demonstrates good discriminative power and stability. These findings advocate for a shift from static haemodynamic monitoring towards a cumulative burden assessment, enabling more precise, long-term risk stratification in the dialysis population.

## Acknowledgments

The authors gratefully acknowledge the Local Ethics Committee, as well as the medical and nursing staff, who contributed to the collection of the data required for this study.

## Statement of Ethics

This study protocol was reviewed and approved by [“*Comitato Etico Lazio Area 2*”], Rif. 6334 Prot 0000/2021. The study was conducted in accordance with the Declaration of Helsinki. Written informed consent to participate in the study was obtained from all participants prior to enrolment. No directly or indirectly identifying information is included in this manuscript or in its online supplementary material (for all online suppl. material, see https://doi.org/10.1159/kbr/adjag007).

## Conflict of Interest Statement

The authors have no conflicts of interest to declare. Sandro Mazzaferro was a member of the journal’s Editorial Board at the time of submission.

## Funding Sources

This study was not supported by any sponsor or funder.

## Author Contributions

Roberto Casale, Silverio Rotondi, Lida Tartaglione and Francesco Pesce: conceptualization. Silverio Rotondi: project administration and supervision. Francesco Pesce: supervision, conceptualization and writing – review and editing. Roberto Casale: methodology, formal analysis, software, visualization and writing – original draft. Francesco Pesce, Roberto Casale and Silverio Rotondi: design, development and testing of the web-based calculator (software). Antonio Bellasi: formal analysis and writing – review and editing. Sandro Mazzaferro, Silvia Lai, Sebastiano Sciarretta, Maria Luisa Muci, and Gianluigi Zaza: investigation, data curation and writing – review and editing. All authors critically reviewed subsequent drafts and approved the final version of the manuscript for submission.

## Data Availability

The clinical data underlying this article are not publicly available due to privacy and ethical restrictions related to patient confidentiality but will be shared on reasonable request to the corresponding author, provided that the request complies with applicable privacy regulations and ethical requirements. The source code for the web-based calculator supporting the findings of this study is publicly available at https://github.com/roberto-casale/CAVI_Web_App. The study was reported in accordance with the Strengthening the Reporting of Observational Studies in Epidemiology (STROBE) guidelines; the completed STROBE checklist is provided as online supplementary material.
